# Nitric Oxide-Induced Dormancy Removal of Apple Embryos Is Linked to Alterations in Expression of Genes Encoding ABA and JA Biosynthetic or Transduction Pathways and RNA Nitration

**DOI:** 10.3390/ijms20051007

**Published:** 2019-02-26

**Authors:** Paulina Andryka-Dudek, Katarzyna Ciacka, Anita Wiśniewska, Renata Bogatek, Agnieszka Gniazdowska

**Affiliations:** Department of Plant Physiology, Warsaw University of Life Sciences-SGGW, Nowoursynowska 159, 02-776 Warsaw, Poland; paulina.andryka@gmail.com (P.A.-D.); anita_wisniewska@sggw.pl (A.W.); renata_bogatek@sggw.pl (R.B.); agnieszka_gniazdowska@sggw.pl (A.G.)

**Keywords:** ABA, jasmonic acid, nitric oxide, RNA nitration, seed dormancy, seed germination

## Abstract

Short-term (3 h) treatment of embryos isolated from dormant apple (*Malus domestica* Borkh.) seeds with NO donors stimulates their transition from dormancy to germination. Seed dormancy is maintained by ABA, while germination is controlled mainly by gibberellins (GAs) and jasmonic acid (JA). NO-induced dormancy removal correlates with low ABA concentration in embryonic axes and reduced embryo sensitivity to ABA. We analyzed the expression of genes encoding key enzymes of ABA degradation (*CYP707A1*, *CYP707A2*), biosynthesis (*NCED3*, *NCED9*), and elements of the ABA transduction pathway (*PYL1*, *PYL2*, *RCAR1*, *RCAR3*, *PP2CA*, *ABI1*, *ABI2*, *SNRK2*, *ABI5*, *AREB3*, *ABF*). A role for JA in the regulation of germination led us to investigate the expression of genes encoding enzymes of JA biosynthesis (*AOS1*, *JMT*, *JAR1*) and the transduction pathway (*COI1*, *MYC2*, *JAZ3*, *JAZ12*). The expression profiles of the genes were estimated in embryonic axes isolated from dormant or NO fumigated apple embryos. The analyzed genes were differentially regulated during dormancy alleviation, the main modifications in the transcription level were detected for *NCED3*, *NCED9*, *CYP707A2*, *RCAR1*, *ABF*, *AOS1*, *JMT*, *JAR1* and *JAZ3*. A regulatory role of NO in the removal of seed dormancy is associated with the stimulation of expression of genes related to ABA degradation, down-regulation of genes responsible for ABA synthesis, an increase of expression level of genes engaged in JA synthesis and modification of the expression of genes engaged in signaling pathways of the hormones. To confirm a signaling role of NO during dormancy breakage, an increased RNA nitration level in embryonic axes was demonstrated.

## 1. Introduction

Seed germination is the first developmental phase in the life cycle of a higher plant. Seed germination *sensu stricto* starts with water uptake by the dry seed and ends at the moment of radicle protrusion, resulting from elongation of the embryonic axis [1]. This process involves the integration of various environmental signals, endogenous hormones and complex communication between cells and seed compartments [2]. In seeds of the majority of plant species, germination is preceded by the removal of dormancy. Dormancyremoval and seed transduction from a dormant state to germination is regulated by the balance of plant hormones, predominantly gibberellins (GAs) and abscisic acid (ABA). Other classical hormones such as ethylene, brassinosteroids and jasmonic acid (JA) form a regulatory network with plant growth regulators e.g., polyamines and other signaling molecules, including reactive oxygen species (ROS) or reactive nitrogen species (RNS) [3,4]. Nitric oxide (NO) belongs to RNS and is considered as a seed germination inducing factor [5,6]. Its role in the antagonistic action of ABA and GA in seed germination is well described in the literature [7,8,9], while ABA-JA-NO interaction is less explored. 

Apple (*Malus domestica* Borkh.) seeds are characterized by deep embryonic dormancy, described as combinational (PY + PD): physical (PY) and physiological (PD) dormancy originating from i) the presence of seed coat impermeable to water and gases; ii) the existence of biochemical barriers inhibiting germination even in the favorable environmental conditions. Dormancy of apple seeds may be overcome by 90 days of long cold (5 °C) stratification [10,11]. A similar physiological consequence (fast, uniform and high germination rate of the embryos) is observed after short term (3 h) fumigation of isolated embryos with NO_x_ or various donors of NO (*S-*nitroso-N-acetylpenicillamine, SNAP or sodium nitroprusside, SNP) [12,13]. The beneficial effect of cold stratification on apple seed germination is due to changes in phytohormonal balance (Reference [11] and references therein). It occurs in the first month of the chilling treatment and is characterized by a decreased ABA content, increased GA concentration accompanied by an increased JA level, the maximum of which is reached when ABA is undetectable [11]. In addition, dormancy removal of apple seeds by cold stratification is associated with fluctuation in ROS generation and stimulation of NO emission in the embryonic axes [10]. Moreover, as demonstrated on *Arabidopsis thaliana* seeds, the positive action of NO on seed dormancy breakage was associated with induction of GA biosynthesis and ABA catabolism [14,15]. Jacobsen et al. [16] indicated that in wheat (*Triticum aestivum* L.) caryopsis NO was required for methyl jasmonate (MeJA) to reduce dormancy and MeJA was needed for NO-dependent regulation of this process. The authors suggested that MeJA and NO do not act sequentially but they probably act interdependently, requiring each other to alleviate seed dormancy. 

The aim of our work was to investigate at the transcription level the ABA-JA-NO interaction during dormancy removal of apple embryos. It is suggested that NO acts as a signaling molecule that triggers the reaction cascade leading to seed transduction from dormant to non-dormant state. Therefore, as the material in this study we have used axes of dormant embryos after 3 h fumigation with vapors of acidified nitrite [12,13]. The choice of this experimental model led us to point at the rapid reaction of dormant tissue to an elevated level of NO in the environment at the early stages of seed re-hydration. To build up a summary of ABA-JA-NO network in the process of dormancy breakage we determined NO impact on transcription of genes encoding crucial enzymes of ABA and JA biosynthesis, ABA catabolism, and elements of ABA and JA transduction pathways. 

Fumigation of apple embryos with NO resulted in both an enhanced formation of superoxide radicals (O_2_^−•^) and an increased emission of NO in the embryonic axes just after treatment [17] indicating the putative formation of peroxynitrite (ONOO^−^). Therefore, to underline that short-term NO exposition of the embryos is sufficient for dormancy loss we have measured the level of RNA nitration in the axes. ONOO^−^ can nitrate guanine and related nucleosides and nucleotides in free form or in DNA or/and RNA. Nitration of guanine results in the formation of 8-nitro-guanine (8-NO_2_-G) [18]. In plants, this reaction occurs relatively rapidly, as in leaves of potato (*Solanum tuberosum* L.) in response to pathogen inoculation; its first maximum was observed within 3 h [19]. Although, several examples of RNA nitration were reported in animals and humans (Reference [20] and references therein), such information in plants is unique. Moreover, the data on alterations in 8-NO_2_-G content in RNA in seeds during dormancy removal could be of great interest because of the idea that oxidative damage of RNA (particularly mRNA), leading to the formation of 8-oxo-guanine (8-oxo-G), acts as a signaling event that tracks seeds toward germination state (Reference [21] and references therein).

Our results indicate that in apple embryos, a shift from dormant into a non-dormant state after short-term NO treatment is linked to the slight accumulation of 8-NO_2_-G in the RNA pool. We have demonstrated that NO-stimulated dormancy breakage of apple embryos is associated with the up-regulation of genes related to ABA degradation, down-regulation of genes responsible for ABA synthesis, the increased expression level of genes engaged in JA synthesis and alterations in the expression of genes engaged in signaling pathways of both hormones.

## 2. Results

### 2.1. Dormancy Removal of Apple Embryos by Short-Term NO Treatment Was Associated with Alterations in the Expression of Genes Encoding Enzymes of ABA Biosynthetic Pathway and ABA Degradation

#### 2.1.1. Short-Term Fumigation with NO Decreased *NCED3* and *NCED9* Transcripts Level in Embryonic Axes of Apple Seeds

**Figure 1:**
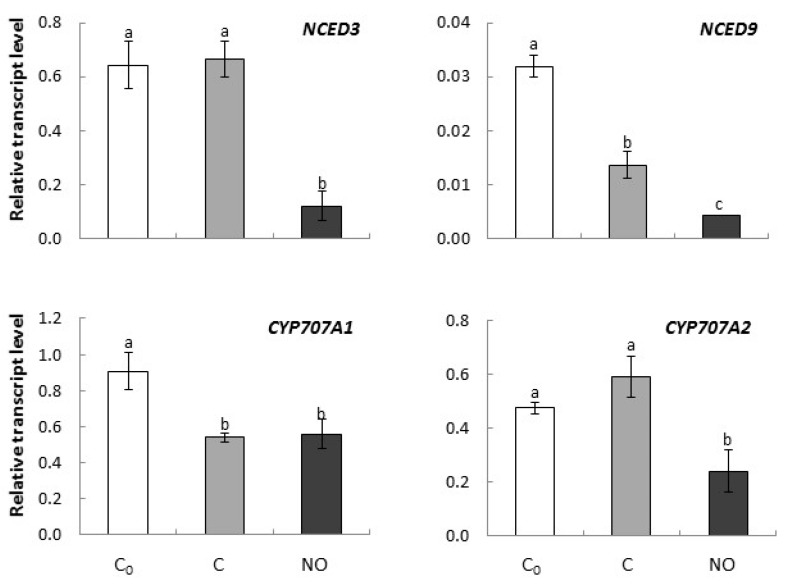
The relative expression level of genes encoding enzymes of ABA biosynthesis (*NCED3*, *NCED9*) and genes encoding enzymes of ABA catabolism (*CYP707A1*, *CYP707A2*) in axes of apple embryos isolated after 24 h of seeds imbibition in water (C_0_), in axes of embryos imbibed in water for 3 h after seed coat removal (C), in axes of embryos fumigated with NO for 3 h (NO). The expression of genes was normalized to *UBI* as a reference gene. Two technical replicates were performed for each of four biological replicates. Error bars represent ± SD, a–c homogenous groups.

The *NCED3* expression level in axes of dormant embryos (after 24 h of seeds imbibition in water, C_0_) did not change as the imbibition was prolonged up to 3 h after the seed coat was removed (C) (Figure 1). Treatment of apple embryos with NO led to drastic 5 fold decrease of the *NCED3* transcript level as compared to dormant embryos (C and C_0_) (Figure 1).

The expression level of *NCED9* was very reduced in all tested plant material (Figure 1) compared to other analyzed genes. After 3 h of prolonged imbibition of dormant embryos, *NCED9* transcript accumulation in axes decreased twice. Fumigation of the embryos with NO resulted in a decrease of the expression level of *NCED9*. *NCED9* transcript accumulation in NO-treated embryos was 10 fold lower than in dormant embryos after 24 h of seed imbibition in water (C_0_) (Figure 1). 

#### 2.1.2. Short-term Fumigation with NO Had no Effect on *CYP707A1* and Decreased *CYP707A2* Transcripts Level in Embryonic Axes of Apple Seeds

The *CYP707A1* expression level declined as water uptake by the embryos was extended (Figure 1). NO did not influence the transcript level of the gene, which was at the range of control after 3 h of imbibition (C).

Prolongation of imbibition of dormant embryos for an additional 3 h (C) did not alter *CYP707A2* transcript level in embryonic axes in comparison to C_0_ (Figure 1). The relative expression level of *CYP707A2* in NO fumigated tissue was twice lower than in non-treated, dormant axes (C) (Figure 1).

### 2.2. Dormancy Removal of Apple Embryos by Short-Term NO Treatment Was Associated with Alterations in the Expression of Genes Encoding Elements of ABA Signal Transduction Pathway

#### 2.2.1. Short-Term Fumigation of Apple Embryos with NO Increased the Expression of *RCAR1* but Did Not Influence the Expression of Other Genes Encoding ABA Co-Receptors (*PYL1*, *PYL2*, *RCAR3*) in Embryonic Axes

**Figure 2:**
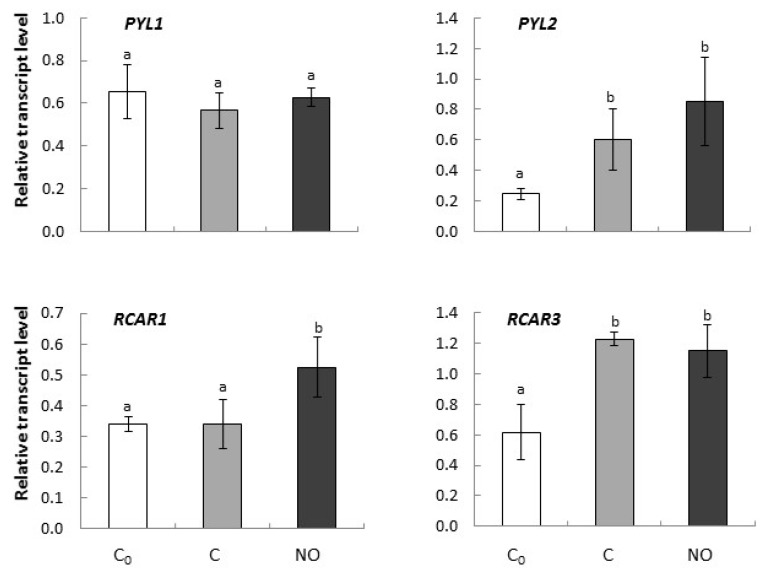
The relative expression level of genes encoding ABA co-receptors (*PYL1*, *PYL2* and *RCAR1*, *RCAR3*) in axes of apple embryos isolated after 24 h of seeds imbibition in water (C_0_), in axes of embryos imbibed in water for 3 h after seed coat removal (C), in axes of embryos fumigated with NO for 3 h (NO). The expression of genes was normalized to *UBI* as a reference gene. Two technical replicates were performed for each four biological replicates. Error bars represent ±SD; a, b homogenous groups.

*PYL1* expression did not differ in the analyzed plant material independently of the treatment and the experiment continuation (Figure 2). *PYL2* was up-regulated by the prolongation of the imbibition period and NO fumigation, although there were no significant differences between the transcript level of *PYL2* in axes of NO-treated embryos and in embryos after prolonged imbibition (C) (Figure 2).

*RCAR1* was significantly up-regulated by NO treatment in comparison to both controls. The transcript level of this gene did not differ in axes of dormant, non-treated embryos (C_0_ and C) (Figure 2). *RCAR3* expression level increased after 3 h of imbibition independently of NO application (Figure 2).

#### 2.2.2. Short-term Fumigation of Apple Embryos with NO Did Not Influence the Expression of Genes (*PP2CA*, *ABI1*, *ABI2*) Encoding Protein Phosphatases PP2Cs and *SnRK2* Encoding Protein Kinase in Embryonic Axes

**Figure 3:**
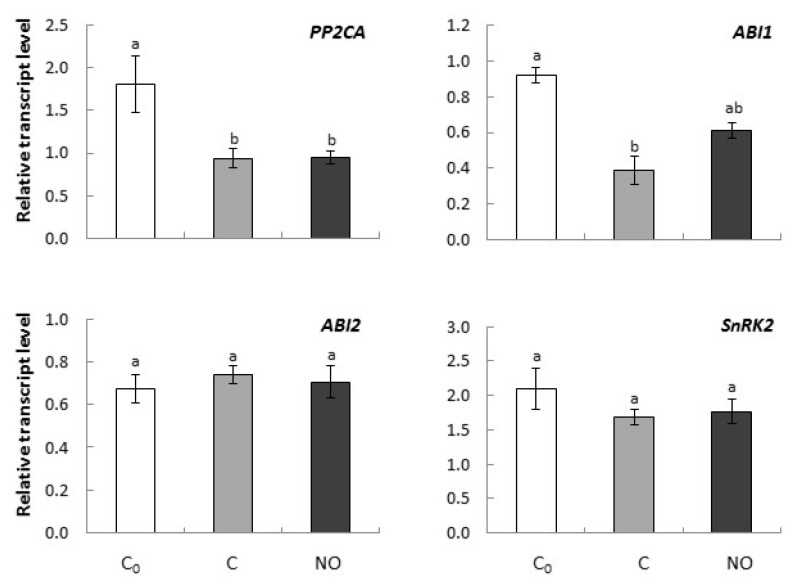
The relative expression level of genes encoding elements of ABA transduction pathway (*PP2CA*, *ABI1*, *ABI2* and *SnRK2*) in axes of apple embryos isolated after 24 h of seeds imbibition in water (C_0_), in axes of embryos imbibed in water for 3 h after seed coat removal (C), in axes of embryos fumigated with NO for 3 h (NO). The expression of genes was normalized to *UBI* as a reference gene. Two technical replicates were performed for each four biological replicates. Error bars represent ± SD; a, b homogenous groups.

The expression level of *PP2CA* in embryonic axes decreased after prolongation of the imbibition period, independently of NO treatment (Figure 3). *ABI1* was down-regulated in embryonic axes as the imbibition period was extended and by NO fumigation. No significant difference was observed in the ABI1 transcript level in axes of NO-treated embryos in comparison to a control (C) (Figure 3). Transcription of *ABI2* and *SnRK2* was not changed as the experiment was performed, and NO fumigation had no impact on the expression level of them (Figure 3). 

#### 2.2.3. Short-term Fumigation of Apple Embryos with NO Prevented Increase of the Expression Level of Gene Encoding ABF While Had No Effect on the Expression of Genes Encoding Other ABA Transcription Factors (ABI5 and AREB3) in Embryonic Axes

**Figure 4:**
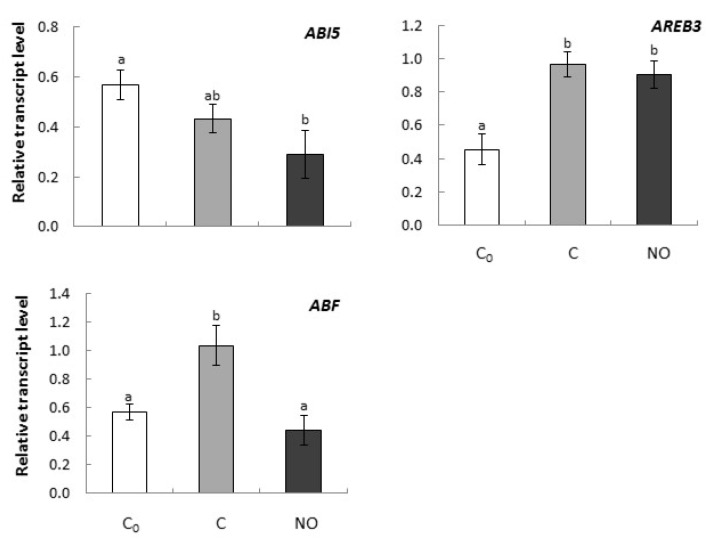
The relative expression level of genes encoding ABA transcription factors (*ABI5*, *AREB3*, *ABF*) in axes of apple embryos isolated after 24 h of seeds imbibition in water (C_0_), in axes of embryos imbibed in water for 3 h after seed coat removal (C), in axes of embryos fumigated with NO for 3 h (NO). The expression of genes was normalized to *UBI* as a reference gene. Two technical replicates were performed for each four biological replicates. Error bars represent ± SD; a, b homogenous groups.

Transcription of *ABI5* in embryonic axes decreased insignificantly as the imbibition period of the embryos was extended for an additional 3 h. The expression level of the gene was lower in axes of NO-treated embryos as compared to C_0_ but not to C (Figure 4). 

Up-regulation of *AREB3* was detected after prolonged imbibition and NO treatment of the embryos (C and NO). The up-regulation of *AREB3* was independent of NO treatment (Figure 4). 

*ABF* up-regulation was observed in axes of dormant embryos (C) after an additional 3 h of water uptake (Figure 4). No up-regulation was observed in embryos after NO application.

### 2.3. Dormancy Removal of Apple Embryos by NO Fumigation Was Linked to Alterations in Expression of Genes Encoding Enzymes of JA Biosynthesis and JA Derivatives Formation

#### Dormancy Removal of Apple Embryos by NO Fumigation was Linked to Up-Regulation of Genes Encoding Enzymes of JA Biosynthesis (AOS1) and MeJA Formation (JMT)

**Figure 5:**
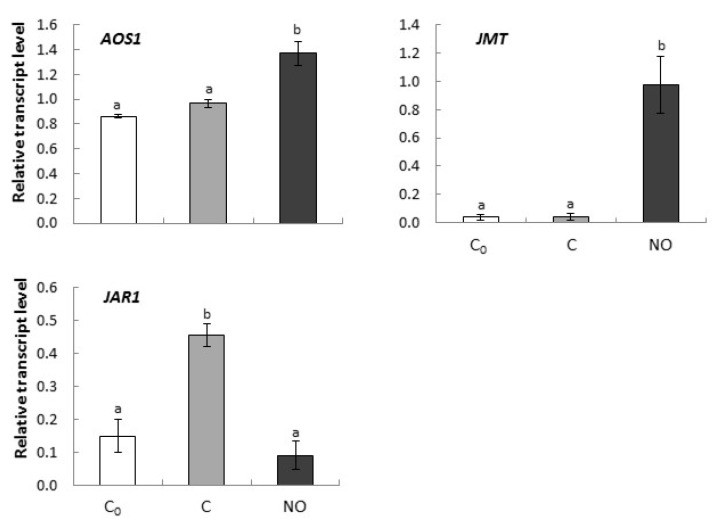
The relative expression level of genes encoding enzymes of JA biosynthesis (*AOS1*), and JA derivatives formation (*JMT*, *JAR1*) in axes of apple embryos isolated after 24 h of seeds imbibition in water (C_0_), in axes of embryos imbibed in water for 3 h after seed coat removal (C), in axes of embryos fumigated with NO for 3 h (NO). The expression of genes was normalized to *UBI* as a reference gene. Two technical replicates were performed for each four biological replicates. Error bars represent ± SD; a, b homogenous groups.

The expression level of *AOS1* in axes of dormant apple embryos did not change as water uptake was extended for an additional 3 h (C) (Figure 5). NO fumigation of the embryos led to up-regulation of *AOS1*, which transcripts were 40% more abundant than in dormant tissue after the extended imbibition (Figure 5). 

The expression level of *JMT* in axes of dormant apple embryos after NO treatment was strongly up-regulated comparing to both 24 h (C_0_) and prolonged imbibition (C) (Figure 5).

*JAR1* up-regulation was observed in axes of dormant embryos after an additional 3 h of water uptake (Figure 5). No increase in *JAR1* expression was observed in embryos after NO application, as compared to C_0_.

### 2.4. Dormancy Removal of Apple Embryos by NO Fumigation Was Linked to Alterations in Expression of Genes Encoding Elements of JA Signal Transduction Pathway

#### Dormancy Removal of Apple Embryos by NO Fumigation Was Linked to Alterations in the Expression of *JAZ3* Encoding Negative Regulator of JA Signal Transduction Pathway

**Figure 6:**
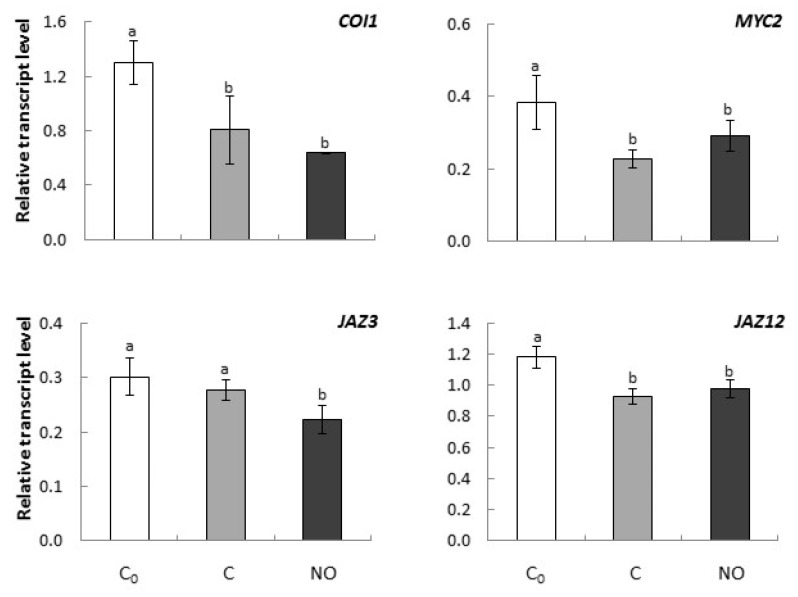
The relative expression level of genes encoding elements of JA transduction pathway (*COI1*, *MYC2*, *JAZ3*, *JAZ12*) in axes of apple embryos isolated after 24 h of seeds imbibition in water (C_0_), in axes of embryos imbibed in water for 3 h after seed coat removal (C), in axes of embryos fumigated with NO for 3 h (NO). The expression of genes was normalized to *UBI* as a reference gene. Two technical replicates were performed for each four biological replicates. Error bars represent ± SD; a, b homogenous groups.

The expression level of *COI1* in embryonic axes was reduced after prolonged imbibition as well as after NO fumigation, but no differences were observed between these conditions (Figure 6).

*MYC2* expression was the highest at the beginning of the experiment (Figure 6). Prolongation of apple embryos imbibition, as well as NO treatment, resulted in a similar decrease of *MYC2* transcript level (Figure 6).

The slight, but statistically significant, decrease in the expression level of *JAZ3* was observed after NO treatment of embryos in comparison to both 24 h and prolonged imbibition (C_0_ and C) (Figure 6). Whereas, *JAZ12* expression slightly declined to the similar level in NO and non-treated embryos imbibed for additional 3 h (C) as compared to the *JAZ12* expression level at the beginning of the experiment (C_0_) (Figure 6).

### 2.5. NO Fumigation of Apple Embryos Increased RNA Nitration Level in Embryonic Axes

To prove that ONOO^−^ is able to induce nitration of RNA in vivo, we measured the level of 8-NO_2_-G in isolated dormant apple embryos treated with SIN-1 (0.3 mM). Exposition of apple embryos to SIN-1 for 3 h resulted in an elevated content of 8-NO_2_-G (Table 1). The RNA nitration level in axes of dormant embryos was independent on imbibition period, and 8-NO_2_-G content was ~73 pg µg^−1^ RNA. Fumigation of the embryos with NO (leading to dormancy breakage) resulted in increased 8-NO_2_-G content in RNA in embryonic axes (Table 1). 

## 3. Discussion

NO is a signaling molecule, regulating several processes in plants, starting from seeds germination to seedlings senescence [22]. Studies performed on a whole-genome reported 71 differentially expressed genes (DEGs) in *A. thaliana* leaves after infiltration with SNP, which was used as NO donor to induce hypersensitive cell death [23]. Transcriptome analysis of *A. thaliana* leaves and roots treated with nitrosoglutathione (GSNO) revealed 3263 DEGs involved in many physiological processes [24]. Microarray analysis of *A. thaliana* roots treated with SNP indicated 422 DEGs (342 up-regulated and 80 down-regulated) involved in plant defense, oxidative stress, signal transduction of ethylene and ABA [25]. Huang et al. [26] performed a transcriptome analysis of NO-responsive genes in upland cotton (*Gossypium hirsutum* L.) leaves. Using SNP (100 and 250 µM) they identified DEGs related to plant hormones biosynthesis and signal transduction pathways, among which there were detected 17 genes related to ABA and five genes related to JA. Therefore, although a lot of evidence on NO impact on genes expression in plants can be found in the literature, detailed information on NO regulation of specified ABA and JA genes in seeds, particularly at the stage of dormancy alleviation, is random and needs to be completed. 

ABA is considered as the main hormone determining seed dormancy, responsible for inhibition of seed germination (Reference [27] and references therein). The concentration of ABA is the highest in mature, dormant seeds, while it declines as dormancy is lost, and transduction from dormancy to germination state is proceeded [28]. 

*CYP707A* family encodes ABA 8′-hydroxylase regulating ABA catabolism in seeds of many plants (Reference [27] and references therein). We were surprised that in axes of apple embryos decrease of *CYP707A2* transcription after 3 h of NO treatment was detected, while *CYP707A1* transcripts were at the same level in dormant (C) and NO-treated tissue. This observation is in contrast to data presented by Liu et al. [15], showing the rapid induction of *CYP707A2* transcription in *A. thaliana* seeds exposed to SNP in the first 6 h of imbibition. This incompatibility may be due to different NO donors used in the experiment (SNP decomposes to NO^+^ and CN^−^) and longer duration of SNP treatment in experiments performed at *A. thaliana* seeds. In *cyp707a1* and *cyp707a3* mutants of this plant species, the rate of ABA catabolism was enhanced by SNP [15]. After prolongation of germination of NO-treated apple embryos, we could expect the increased transcription of both *CYP707A* genes because dormancy removal by NO is linked to reduced ABA content in the seeds and their sensitivity to ABA [12,29,30]. 

ABA cellular level is also controlled by its biosynthesis. The enzyme nine-cis-epoxycarotenoid dioxygenase (NCED) catalyzes the key step in ABA biosynthesis. *NCED* expression in response to environmental stresses is so rapid that NCED activity is considered as the rate-limiting step in ABA biosynthesis [27]. The overproduction of NCED6 and NCED9 occurs in embryogenesis and regulates seed development and dormancy. In *A*. *thaliana*, *NCED6* and *NCED9* are highly expressed in developing seeds, and mutational analysis of these two genes indicated their role in seed dormancy [31]. In axes of apple seeds expression of *NCED3* and *NCED9* was strongly inhibited by short-term NO fumigation, suggesting a negative effect of NO on ABA synthesis, and consequently weakening dormancy. A detailed analysis of transcriptome profiling in *A. thaliana* leaves after infiltration with CysNO indicated down-regulation of genes encoding enzymes of the ABA biosynthetic pathway, such as *KAO1* (encoding an ent-kaurenoic acid hydroxylase) or *NCED4* [32]. While in *A. thaliana* leaves, *NCED3*, in contrast to apple embryos, was up-regulated [32]. Dissimilarities in the expression of *NCED3* in *A. thaliana* leaves and axes of apple embryos after NO exposition may be due to NO donor and particularly tissue specificity but the final effect of NO activity remains the same in both experiments and results in an reduction of ABA content and removal of seed dormancy.

Perception of the ABA signal in plants depends on receptors of the PYR/PYL/RCAR family [33]. ABA binding by PYR/PYL/RCAR and interaction of the complex ABA-receptor with type 2C protein phosphatases (PP2Cs) leads to PP2Cs inhibition. This inactivation of PP2C results in activation of SNF1-related protein kinase 2 (SnRK2), which promotes the activity of transcription factors e.g., ABI5. Transcripts level of *PYL1*, *PYL2*, *RCAR1* and *RCAR3* in axes of apple embryos increased or did not change as imbibition of the embryos was extended. Fumigation of the apple embryos with NO did not drastically modify the transcription of analyzed genes with exception of *RCAR1*. Similarly, in *A. thaliana* transcription of *RCAR1* in contrast to *RCAR3* was constant or slightly increased upon various conditions [34]. In addition, 50% inhibition of PP2Cs by receptor complex containing RCAR1 instead of RCAR3 occurs at a higher ABA level. No effect of NO on the expression of genes encoding ABA co-receptors may be explained by the reduction of ABA content and a lack of necessity of triggering of the ABA transduction pathway. Confirmation of this hypothesis may also be a lack of induction of ABA-responsive genes like those analyzed in this work; genes encoding transcription factor or in contrast the down-regulation of one of them (*ABF*). 

Posttranslational modifications (PTMs) of proteins (nitration or *S*-nitrosylation) are accepted to be markers of NO or other RNS action [35,36]. To date, well-characterized examples of NO dependent PTMs in plants include the phytohormone biosynthetic enzymes, their receptors and elements of signal transduction pathways [35]. Castillo et al. [37] demonstrated specific nitration patterns of different PYR/PYL/RCAR proteins. They found that several members of the ABA co-receptor family were inactivated through NO-mediated nitration of Tyr residues.

In axes of apple embryos the expression of genes encoding PP2Cs (*PP2CA* and *ABI1*)—members of the ABA signal transduction pathway decreased as the imbibition period was prolonged but, in general, NO had no effect on their transcription. The transcript level of *ABI2* did not differ in axes of dormant (C) and NO-fumigated embryos (NO), nor were changes noticed independently of the imbibition period of the embryos (C_0_ compared to C and NO). It can imply that both the duration of water uptake and NO signaling do not lead to any modification of PP2Cs genes expression. Germination of *A. thaliana* seeds of the *pp2c5* mutant on ABA-containing medium was significantly increased compared to WT seeds, indicating that the *pp2c5* mutant displayed partial insensitivity to ABA [38]. It remains likely as ABA content and tissue sensitivity to ABA decline in apple embryos after NO fumigation [12], the more the following element of the ABA transduction pathway seems to be under RNS control.

The next step of ABA signal transduction cascade depends on the SnRK2 group of kinases. SnRK2 proteins were shown to be modified post-translationally by *S*-nitrosylation which leads to their inactivation [39]. The lack of influence of the short-term NO treatment on the expression of *SnRK2* in axes of apple embryos confirms the supposition that regulation of this step of ABA signal transduction by NO occurs rather *via* PTMs than at transcriptomic level. Declined activity of SnRK2 due to *S*-nitrosylation may lead to inactivation of ABI5. Moreover, it is suggested that regulation of SnRK may occur also by Mitogen-Activated Protein Kinase 3 and 6, which the activity and transcription of the genes are ROS-mediated [40]. Dormancy removal of apple embryos by NO and germination process are connected to elevated production of ROS, mainly H_2_O_2_ [17].

ABI5 is a key regulator of the ABA transduction pathway during seed germination. *ABI5* encodes transcription factor that regulates e.g., a subset of late embryogenesis abundant (LEA) genes during embryogenesis. In *A. thaliana*, in NO deficient seeds (seeds of mutants *nia1nia2noa1-2* characterized by restriction in NO biosynthetic pathways), strengthened dormancy was observed, accompanied by hypersensitivity to ABA [41], which was explained by over-accumulation of ABI5 [42]. Two days or longer treatment of *A. thaliana* seeds with SNAP or GSNO led to a decrease in the *ABI5* transcript level [42]. Similarly, in axes of apple embryos, NO treatment also resulted in a slight down-regulation of *ABI5*, suggesting a decline in the ABA response, which is typical for seeds at the stage of dormancy breakage. *ABF* is an ortholog of *ABI5*. NO fumigation led to a decrease of the transcript level of *ABF* as compared to control after 3 h of additional water uptake (C), although in contrast to *ABI5*, its expression increased during imbibition of dormant apple embryos (C compared to C_0_). It can suggest that NO prevents *ABF* expression. Up-regulation of *ABF* during prolonged imbibition of apple embryos is in agreement with data reported for dormant wheat grains showing an increased expression of *TaABF1* [43]. Moreover, it was shown that NO stimulates the deprivation of the ABI5 protein by *S*-nitrosylation at Cys-153. This PTM targets ABI5 into proteasome degradation [42]. AREB3 is ABA-responsive element binding protein 3. It binds to the ABA-responsive element (ABRE) located in gene promoter sequences. The predicted partner of AREB3 is SnRK, thus, modifications in SnRK may influence AREB3 function. ABRE-binding proteins (AREBs)/ABRE-binding factors (ABFs) can bind to ABRE elements, resulting in the upregulation of ABA-responsive genes. The transcript level of *AREB3* in axes of dormant apple embryos increased as the duration of imbibition period was prolonged, but no effect of NO fumigation was noticed. It may indicate that NO-dependent regulation of ABA-responsive genes in apple seeds occurs *via* alterations at the upstream level of the hormone signaling cascade.

The role of jasmonates (JA and its derivatives methyl jasmonate (MeJA) or jasmonyl-isoleucine (JA-Ile)) in plant physiology includes not only regulation of the defense against herbivores and pathogens but also many developmental processes [44]. Jasmonates were shown to stimulate germination of dormant seeds of plants belonging to e.g. Rosaceae family such as pear (*Purus communis* L.) [45] or apple [46], while they inhibited germination of non-dormant seeds. JA content in apple embryos increased during dormancy removal by cold stratification (it had two maximum; the first after 30 days of the treatment and the second, visibly smaller after around 70 days) (Reference [11] and references therein), suggesting its essential action in seed transduction from a dormant to non-dormant state. JAs is synthesized from α-linoleic acid. Allene oxidase (AOS) is one of the key enzymes of conversion of α-linoleic acid into 12-oxo-phytodienoic acid (OPDA)—the precursor of JA (Reference [44] and references therein). In our experiment, in axes of apple embryos, the transcript level of *AOS* increased after NO short-term fumigation in about 40% pointing at stimulation of JA synthesis. This result is in agreement with the up-regulation of *TaAOS1* and *TaAOS2* in germinating, stratified wheat grains [47] and analysis of DEGs in *A. thaliana* leaves after application of CysNO, which indicated an accumulation of *AOS* transcripts [32]. Importance of JA in the regulation of germination of dormant seeds was confirmed by Jacobsen et al. [16]. They identified 82 DEGs (the expression of the genes was changed twofold or more) in dormant and dormant MeJA treated wheat caryopsis (22 up- and 60 genes down-regulated). Among the up-regulated genes, it was found gene encoding germin-like protein 4, which in barley (*Hordeum vulgare* L.) was expressed mostly at post germination stage - the early stage of young seedling development or after treatment with H_2_O_2_ [48]. 

*JMT* encodes an *S*-adenosyl-l-methionine:jasmonic acid carboxyl methyltransferase that catalyzes the formation of MeJa from JA. In axes of NO fumigated apple embryos, *JMT* expression level increased drastically as compared to dormant tissue (both C_0_ and C). In contrast, the up-regulation of *JAR1* characteristic for prolonged imbibition was prevented by NO treatment. JAR1 catalyzes the synthesis of jasmonates-amino acid conjugates by adenylation and usually uses Ile as a conjugating amino acid to form JA-Ile. Based on the presented data, we can suggest that during the NO-induced transition of apple embryos from a dormant to non-dormant state, the formation of MeJA is more important than JA-Ile synthesis. 

The JA signal transduction pathway requires the COI1 receptor (coronatine insensitive 1) that belongs to the F-box proteins, a part of the E3 ubiquitin ligase SCF-complex. This complex is involved in the degradation of the Jasmonate Zim domain (JAZ)—JA signaling pathway repressors [49]. The regulatory elements of promoters of JA-regulated genes are recognized by MYC2 or a novel interactor of JAZ (NINJA) (transcriptional activators), which is released as a result of JAZ degradation and can bind to DNA sequences. JA binding to the COI1 and JAZ complexes results in the degradation of JAZ protein and the release of transcription factors (MYCs). The transcription factors activate the expression of JA responsive genes and trigger a downstream response [49].

In axes of dormant apple embryos, the *COI1* expression level decreased as the imbibition period was prolonged. NO application did not change the expression level of this gene compared to prolonged imbibition (C_0_). It may suggest a relatively lower abundance of the COI1 receptor protein in embryonic axes at the initial stage of dormancy alleviation, resulting in a decreased sensitivity of the tissue to JA, which can be compensated/balanced by putative stimulation of hormone synthesis and/or alterations in expression of *JAZs* and/or *MYCs*. The *JAZ12* expression level did not differ in axes of dormant and NO-fumigated embryos, whereas *JAZ3* was slightly but statistically significant down-regulated in NO-treated tissue. Proteolysis of JAZ proteins activates not only JA transcription factors MYC, but also ethylene response factor 1 (ERF1) (Reference [50] and references therein), which could be a critical step in dormancy removal of apple embryos by NO, since we observed enhanced ethylene emission from the embryos after NO fumigation [12].

The ABA-JA interaction in the regulation of germination observed in our work differs fundamentally from that one described in *A. thaliana*, due to use of non-dormant *Arabidopsis* seeds in the tests. In contrast to dormant apple embryos, germination of which was stimulated by jasmonates [46], in non-dormant *A. thaliana* seeds a synergistic effect has been observed, when ABA and JAs were combined to inhibit germination. In a stratified *A. thaliana* ecotype, Col-0 seeds the application of jasmonates to the imbibition medium enhanced ABA-induced inhibition of germination [51,52]. In experiments performed with *cra* mutants (*coi1-16 resistant to aba*) with declined sensitivity to ABA, the same authors confirmed cross-talk of JA and ABA and demonstrated that alterations in a signal transduction pathway of one hormone can affect the sensitivity of the plant to another hormonal signaling pathway.

The clear explanation of the role of JA and ABA in dormancy release by cold stratification focused on *NCED*s central position was done for wheat seeds [47]. We can imagine a similar scheme of NO mediated JA-ABA interaction in dormancy removal of apple embryos at transcriptomic level, although some additional data are necessary (e.g., quantification of jasmonates contents, which we expect to be alike during cold stratification) for its verification. 

The presented data demonstrate evidence of RNA nitration in plant material, as 3 h of fumigation with vapors of acidified nitrite or SIN-1 treatment of apple embryos led to an elevated content of 8-NO_2_-G in the RNA pool in embryonic axes. This observation confirms an earlier report of Izbiańska et al. [19], describing RNA and mRNA nitration in response to pathogen inoculation. They suggested that RNA nitration may alter RNA function and metabolism similarly as oxidation of RNA resulting in the formation of 8-oxo-G. Gene transcripts stored in dry mature seeds represent residuals of mRNAs from seed developmental processes that serve as substrates for the synthesis of proteins during imbibition (Reference [53] and references therein). Oxidation of mRNA in sunflower (*Helianthus annuus* L.) seeds during after-ripening was not a random process, altering the translation of selected proteins [54]. The authors proposed that targeted mRNA oxidation acts as a signal for transition of the seeds into germination (non-dormant) stage in the early phases of seeds imbibition. Moreover, they emphasized that transcripts identified as main players in seed dormancy regulation and related metabolic or signaling pathways, particularly for hormones or ROS are preferential targets of oxidation. In sunflower seeds, transcripts of gene encoding protein phosphatase 2C PPH1 (PP2C PPH1) showed a strong increase in oxidation level during dormancy alleviation by after-ripening [54]. PP2Cs are members of the ABA transduction cascade and oxidation of their transcripts could result in inhibition of translation of the corresponding protein. Although in our experiment no changes in the *PP2CA* expression level were detected after NO treatment, we cannot exclude that its transcript could be preferentially nitrated, leading to declining sensitivity of the embryos to ABA. Therefore, we can suspect that action of NO as the dormancy removing factor in our experimental model could be associated rather to nitro-oxidative modification of RNA resulting in the formation of 8-NO_2_-G or 8-oxo-G than changes in genes expression level. To verify our suggestion additional research focused on nitration and/or oxidation of mRNA and identification of nitrated/oxidized transcripts are necessary.

## 4. Materials and Methods

### 4.1. Experimental Material

The experiments were performed on apple seeds (*Malus domestica* Borkh.), Antonówka variety, obtained from commercial orchards from Grójec district (Mazovian voivodeship, Poland). Seeds were isolated from apple fruits, dried at room temperature, and stored in dark glass containers at 4 °C. 

Dormancy breakage was achieved by fumigation of embryos isolated from dormant seeds with NO (NO). The control group consisted of embryos isolated from dormant seeds non-treated with NO (C). 

#### Fumigation of Dormant Apple Embryos with NO

Prior to embryos isolation, apple seeds were imbibed for 24 h in distilled water at room temperature. Embryos were isolated manually by removing seed coat and endosperm (C_0_).

Fumigation with NO was done using vapors of acidified NaNO_2_ according to Gniazdowska et al. [17]. Approximately 50 embryos were placed in a 500 mL glass container, on filter paper moistened with 50 mM K-phosphate buffer, pH 7.0. Next, a beaker with 5 mL of 2 mM NaNO_2_ was put in the container and 5 mL of 0.2 M HCl was added to the beaker. Then, the container was tightly closed. Embryos were exposed to NO_x_ vapours for 3 h, in the light, at room temperature. After 3 h, the embryos (NO) were rinsed with distilled water and transferred to glass Petri dishes (150 mm) on filter paper moistened with distilled water. Twenty embryos were placed on each dish. 

The control group (C) consisted of isolated dormant apple embryos imbibed in 50 mM K-phosphate buffer, pH 7.0 and exposed to light for 3 h, similarly to the NO-treated embryos. After, the embryos were rinsed with distilled water and transferred to glass Petri dishes (150 mm) on filter paper moistened with distilled water. Twenty embryos were placed on each dish. Description of the abbreviations of the plant material used in the experiment: C_0_—embryonic axes isolated from dormant seeds imbibed for 24 h in distilled water; C—embryonic axes isolated from embryos imbibed for 3 h after removal of seed coat (non-treated dormant embryos); NO—embryonic axes isolated from embryos treated with NO for 3 h. The plant material was frozen in liquid nitrogen and stored at −80 °C until use. 

### 4.2. Analysis of Gene Expression

#### 4.2.1. RNA Isolation from Axes of Apple Embryos

Total RNA isolation was performed according to Reference [55]. Frozen embryonic axes (100 mg) were ground in a mortar in liquid nitrogen and homogenized in 1 mL of guanidinium thiocyanate/phenol buffer, containing 4 M guanidinium thiocyanate, 0.025 M sodium citrate pH 7.0, 2 M sodium acetate pH 4.0, 0.5% *N*-Lauroylsarcosine sodium salt mixed with aqua phenol at 1:1 (*v*/*v*) and chloroform. After, RNA was precipitated with isopropanol in the presence of the mixture 0.8 M sodium citrate and 1.2 M sodium chloride. Precipitated RNA probes were centrifuged, pellets were washed thrice with 75% ethanol and dissolved in RNase-free water. RNA quantity and quality were checked using the NanoDrop ND2000 spectrophotometer (Thermo Scientific, Wilmington, DE, USA) (260 nm and 280 nm) and 1% agarose gel electrophoresis for approximately 30 min at 90 V. Samples were diluted to a 1 µg·µL^−1^ concentration of nucleic acids and residual DNA was removed using DNase I (Thermo Scientific, EN0523). RNA samples were stored at −80 °C until needed for the cDNA synthesis. 

#### 4.2.2. RT-PCR Conditions

Semi-quantitative Reverse Transcription-Polymerase Chain Reaction (RT-PCR) analysis was used to assess changes in the level of transcript accumulation of the genes. The synthesis of a single cDNA strand was done using a RevertAid^TM^ First Strand cDNA Synthesis Kit (Thermo Fisher Scientific, K1622) according to manufacturer’s instruction. 

The specific primers (Table 2) were designed using Primer3Plus software (http://www.bioinformatics.nl/cgi-bin/primer3plus/primer3plus.cgi). Nucleotide sequences to design the primers were selected on the base of a comparative analysis of nucleotide sequences of *A. thaliana*, available from the National Center of Biotechnological Information NCBI database (http://www.ncbi.nlm.nih.gov/), and of apple genome sequences, available from the Genome Database for Rosaceae (https://www.rosaceae.org/). The degree of similarity between chosen homologous genome sequences of these two species and of their corresponding mRNA sequences was determined using ClustalW software (http://www.ebi.ac.uk/) and verified using the BLASTN algorithm, available on the NCBI website.

The PCR mixture contained 1× DreamTaq buffer, 0.2 mM dNTPs, 1 µM primers, 0.2 U DreamTaq Polymerase (Thermo Scientific, EP0701) and 1 μL cDNA. The gene encoding ubiquitin (*UBI* F: 5′-TTGATCTTTGCTGGGAAACAG-3′ and R: 5′-CACCACCATCATTCAACACC-3′) was used as a reference nucleotide sequence. The optimal number of PCR cycles was determined for each of the primer pairs (Table 2). PCR products were separated using electrophoresis in 1.5% agarose/TBE gels (100 mM Tris, 83 mM boric acid, 1 mM EDTA, pH 8.0) containing ethidium bromide, in the Midi Horizontal Unit device (Sigma-Aldrich, Cambridge, England, UK). All PCRs were performed using the thermal cycler (GeneAmp PCR System 9700) and visualized using transilluminator UV (Gel Logic 200, Kodak, Rochester, NY, USA). PCR products were densitometrically quantified using the Kodak Molecular Imaging Software and normalized to *UBI* expression level. The transcript level of *UBI* was expressed as 1. Experiments were performed in four biological and two technical repetitions.

### 4.3. Measurement of Nitrated RNA Content

Total RNA was isolated using RNAzol^®^RT (Sigma, R4533, St. Louis, MO, USA), according to the manufacturer’s guideline. Analysis of nitrated RNA (8-NO_2_-G) level was done using enzyme immunoassay (OxiSelect™ Nitrosative DNA/RNA Damage ELISA Kit; Cell Biolabs INC., STA-825, San Diego, CA, USA). For this purpose, a 96-well protein-binding plate was coated with 100 μL of 8-NO_2_-G-BSA conjugate and incubated overnight at 4 °C. Thereafter, the solution of the 8-NO_2_-G conjugate was removed and the plate was washed with 1× PBS buffer and dried with paper towel. The blocking of the plate was done using 200 μL of assay diluent per each well for 2 h at room temperature. After the assay diluent was removed, 50 µL of sample (250–300 µg of total RNA) was added and the plate was incubated for 10 min at room temperature on an orbital shaker. In parallel, the standard curve was prepared using a diluted 8-NO_2_-G standard in the concentration range of 0 ng mL^−1^ to 1000 ng mL^−1^.

Immunolabelling was carried out by addition of 50 μL of anti-8-NO_2_-G antibody at recommended dilution for 1 h. After wash steps with 1× wash buffer, 100 μL of diluted Secondary Antibody, HRP Conjugate was added. After 1 h of incubation on an orbital shaker, the solution was removed and microwell strips were washed 3 times with 1× wash buffer. To start the enzymatic reaction, 100 μL of substrate solution was added and the plate was incubated at room temperature on an orbital shaker until the color of the solution changed. The reaction was stopped by adding 100 μL of stop solution. The absorbance was measured at 450 nm with a microplate reader (Sunrise, Tecan, Männedorf, Switzerland). Experiments were done in four biological replicates, in two technical repetitions.

### 4.4. Statistical Analysis

Data were analyzed using Statistica Software. Mean differences were calculated and homogenous groups were evaluated using Tukey’s HSD post-hoc test. Standard deviation (±SD) was also provided to indicate the variations associated with the particular mean values. 

## Figures and Tables

**Table 1:** RNA nitration measured as concentration of 8-NO_2_-G (pg·µg^−1^ RNA) in axes of apple embryos isolated after 24 h of seeds imbibition in water (C_0_), in axes of embryos imbibed in water for 3 h after seed coat removal (C), in axes of embryos fumigated with NO for 3 h (NO), quantification of nitrated RNA measured as 8-NO_2_-G concentration in embryos isolated from seed coat after treatment with 0.3 mM SIN-1 in light conditions for 3 h (SIN-1).

Plant Material	Concentration of 8-NO_2_-G (pg µg^−1^ RNA)
C_0_	72.5 ± 3.2 ^a^
C	73.0 ± 3.9 ^a^
NO	84.2 ± 3.6 ^b^
SIN-1	103 ± 4.1 ^c^

Two technical replicates were performed for each four biological replicates. Mean values ± SD; a–c homogenous groups.

**Table 2:** Accession number, source of sequence and primers sequence of genes analyzed by semiquantitative PCR.

Gene	Accession Number	Nucleotide Database	Forward Primer	Reverse Primer
*NCED1*	XM_008380174.2	NCBI	5′- ATCGGCTCCTGCATGACA -3′	5′- CGAGAGCCAAATAAGTGAAC -3′
*NCED3*	XM_008384748.2	NCBI	5′- AGAAGCATATCTACGGTGAC -3′	5′- AGCGCTTATAAACGTTCCATG -3′
*CYP707A1*	AB593330.1	NCBI	5′- CTGGCATTGAAGCATTGGT -3′	5′- TCCTTGCCAAGAGAGCTT -3′
*CYP707A2*	AB593331.1	NCBI	5′- TGCAAGAGATGAAGAGGTATG -3′	5′- GAGAAGCTTCCTTGCCTTC -3′
*PYL1*	KX019762.1	NCBI	5′- GCACTTCATCCGGAGCTGT -3′	5′- GACAACAGTATCAGCGAAGAG -3′
*PYL2*	XM_008379295.2	NCBI	5′- CCTCACAGATACAAGCACT -3′	5′- CTCATTGACCGAAGTAACAG -3′
*RCAR3*	MDP0000191830	GDR	5′- CAAGTTGCTGAGAACAGTGA -3’	5′- CTATCAACGAATCTGTCAACTC -3′
*RCAR1*	MDP0000434532	GDR	5′- CTCTCGTCCGGTACATCA-3′	5′- GAAGAGTAGTTCCTAAGTCTGT-3′
*ABI1*	MDP0000893203	GDR	5′- TGTGGCGATTCGAGAGCA -3′	5′- CTGGATCAGGTATAATCCATGG -3′
*ABI2*	MDP0000647467	GDR	5′- GATGAATGCCTCGTATTAGC -3′	5′- GAGCTTTCAGATCCACCA-3′
*PP2C*	XM_008358502.2	NCBI	5′- GATCGTCGTGGCTAACTGC -3′	5′- GTCGCTCGCCAGGATCAG -3′
*SNRK2*	KJ563286.1	NCBI	5′- AGATTGCAGATGTATGGTC -3′	5′- GCTTTGCATGGGTTGATC-3′
*AREB3*	MDP0000273211	GDR	5′- GTAGGTGCTGGAGCTATGAT -3′	5′- TGTATATGCCTGCTTCCTTG -3′
*ABF*	MDP0000701734	GDR	5′- GAGCTGCAGAACACCATTG -3′	5′- TGTATATGCCTGCTTCCTTG -3′
*AOS1*	XM_008366758.2	NCBI	5′- CGCATCCAGAAATACCAGTCA -3′	5′′- GCTTCAGCTTGTCGTGCT -3′
*JMT*	XM_008378987.2	NCBI	5′- GTTGCTCATCTGGACCAA - 3′	5′- TCAGTTGGTTGTAGAATGCC -3′
*JAR1*	XM_017327397.1	NCBI	5′- GTATTGCCATCTCTTGTGTG -3′	5′- ACAGCTGATCGGATTGATG - 3′
*COI1*	XM_008394693.2	NCBI	5′- GTGTCGTTGGTGTGCAAG -3′	5′- CAGATTGAACATCGCCGC -3′
*JAZ3*	MDP0000243322	GDR	5′- TGACTATTTCAACTGCTGATGC-3′	5′- GATTGGAGAACTGGAGAACTC-3′
*JAZ12*	KU179650.1	NCBI	5′- GAGACACTCTCTTCAGCG-3′	5′- TGAGTTTCTTCCTGAACCATG-3′
*MYC2*	NM_001328944.1	NCBI	5′- CGAACAAGAGTACCGCAAG -3′	5′-GTCGGAACGCAAACCATA - 3′

## Abbreviations

ABF	ABA-Responsive Elements Binding Factor
ABI	Abscisic Acid Insensitive
ABI5	ABA Insensitive 5
AOS	Allene Oxide Synthase
AREB	ABA Response Element Binding Factor
COI	Coronatine Insensitive 1
CYP707	ABA 8’-hydroxylase
GA	Gibberellins
GDR	The Genome Database for *Rosaceae*
JA	Jasmonic Acid
JAR1	Jasmonate-Resistant 1
JAZ	Jasmonate ZIM-domain
JMT	Jasmonate O-methyltransferase
NO	Nitric Oxide
NCED	9-cis-Epoxycarotenoid Dioxygenase
PP2C	Protein Phosphatase 2C
PYL	Pyrabactin resistance 1-like
PYR1	Pyrabactin resistance 1
RCAR	Regulatory Component ABA Receptor 1
RNS	Reactive Nitrogen Species
ROS	Reactive Oxygen Species
SIN-1	3-Morpholinosydnonimine
SNP	Sodium Nitroprusside
SNAP	*S*-nitroso-N-acetylpenicillamine
SnRK2	SNF1-Related Protein Kinase 2

## References

[1] Nonogaki H. (2014). Seed dormancy and germination emerging mechanisms and new hypotheses. Front. Plant Sci..

[2] Nonogaki H. (2017). Seed biology updates–highlights and new discoveries in seed dormancy and germination research. Front. Plant Sci..

[3] Kucera B., Cohn M.A., Leubner-Metzger G. (2005). Plant hormone interactions during seed dormancy release and germination. Seed Sci. Res..

[4] Krasuska U., Ciacka K., Gniazdowska A. (2017). Nitric oxide-polyamines cross-talk during dormancy release and germination of apple embryos. Nitric Oxide.

[5] Krasuska U., Ciacka K., Andryka-Dudek P., Bogatek R., Gniazdowska A., Gupta K.J., Igamberdiev A.U. (2015). “Nitrosative Door” in seed dormancy alleviation and germination. Reactive Oxygen and Nitrogen Species Signaling and Communication in Plants, Signaling and Communication in Plants, Vol. 23.

[6] Šírová J., Sedlářová M., Piterková J., Luhová L., Petřivalský M. (2011). The role of nitric oxide in the germination of plant seeds and pollen. Plant Sci..

[7] Arc E., Sechet J., Corbineau F., Rajjou L., Marion-Poll A. (2013). ABA crosstalk with ethylene and nitric oxide in seed dormancy and germination. Front. Plant Sci..

[8] Arc E., Galland M., Godin B., Cueff G., Rajjou L. (2013). Nitric oxide implication in the control of seed dormancy and germination. Front. Plant Sci..

[9] Sanz L., Albertos P., Mateos I., Sánchez-Vicente I., Lechón T., Fernández-Marcos M., Lorenzo O. (2015). Nitric oxide (NO) and phytohormones crosstalk during early plant development. J. Exp. Bot..

[10] Dębska K., Krasuska U., Budnicka K., Bogatek R., Gniazdowska A. (2013). Dormancy removal of apple seeds by cold stratification is associated with fluctuation in H_2_O_2_, NO production and protein carbonylation level. J. Plant Physiol..

[11] Lewak S. (2011). Metabolic control of embryonic dormancy in apple seed: Seven decades of research. Acta Physiol. Plant..

[12] Gniazdowska A., Krasuska U., Bogatek R. (2010). Dormancy removal in apple embryos by nitric oxide or cyanide involves modifications in ethylene biosynthetic pathway. Planta.

[13] Krasuska U., Ciacka K., Orzechowski S., Fettke J., Bogatek R., Gniazdowska A. (2016). Modification of the endogenous NO level influences apple embryos dormancy by alterations of nitrated and biotinylated protein patterns. Planta.

[14] Bethke P.C., Libourel I.G.L., Aoyama N., Chung Y.-Y., Still D.W., Jones R.L. (2007). The *Arabidopsis* aleurone layer responds to nitric oxide, gibberellin, and abscisic acid and is sufficient and necessary for seed dormancy. Plant Physiol..

[15] Liu Y., Shi L., Ye N., Liu R., Jia W., Zhang J. (2009). Nitric oxide-induced rapid decrease of abscisic acid concentration is required in breaking seed dormancy in *Arabidopsis*. New Phytol..

[16] Jacobsen J.V., Barrero J.M., Hughes T., Julkowska M., Taylor J.M., Xu Q., Gubler F. (2013). Roles for blue light, jasmonate and nitric oxide in the regulation of dormancy and germination in wheat grain (*Triticum aestivum* L.). Planta.

[17] Gniazdowska A., Krasuska U., Czajkowska K., Bogatek R. (2010). Nitric oxide, hydrogen cyanide and ethylene are required in the control of germination and undisturbed development of young apple seedlings. Plant Growth Regul..

[18] Jones L.H. (2012). Chemistry and biology of biomolecule nitration. Chem. Biol..

[19] Izbiańska K., Floryszak-Wieczorek J., Gajewska J., Meller B., Kuźnicki D., Arasimowicz-Jelonek M. (2018). RNA and mRNA nitration as a novel metabolic link in potato immune response to *Phytophthora infestans*. Front. Plant Sci..

[20] Wurtmann E.J., Wolin S.L. (2009). RNA under attack: Cellular handling of RNA damage. Crit. Rev. Biochem. Mol. Biol..

[21] El-Maarouf-Bouteau H., Meimoun P., Job C., Job D., Bailly C. (2013). Role of protein and mRNA oxidation in seed dormancy and germination. Front. Plant Sci..

[22] Baudouin E., Hancock J.T. (2014). Nitric oxide signaling in plants. Front. Plant Sci..

[23] Polverari A., Molesini B., Pezzotti M., Buonaurio R., Marte M., Delledonne M. (2003). Nitric oxide-mediated transcriptional changes in *Arabidopsis thaliana*. Mol. Plant-Microbe Interact..

[24] Begara-Morales J.C., Sánchez-Calvo B., Luque F., Leyva-Pérez M.O., Leterrier M., Corpas F.J., Barroso J.B. (2014). Differential transcriptomic analysis by RNA-seq of GSNO-responsive genes between *Arabidopsis* roots and leaves. Plant Cell Physiol..

[25] Parani M., Rudrabhatla S., Myers R., Weirich H., Smith B., Leaman D.W., Goldman S.L. (2004). Microarray analysis of nitric oxide responsive transcripts in *Arabidopsis*. Plant Biotechnol. J..

[26] Huang J., Wei H., Li L., Yu S. (2018). Transcriptome analysis of nitric oxide-responsive genes in upland cotton (*Gossypium hirsutum*). PLoS ONE.

[27] Rodríguez-Gacio Mdel C., Matilla-Vázquez M.A., Matilla A.J. (2009). Seed dormancy and ABA signaling: The breakthrough goes on. Plant Signal. Behav..

[28] Nambara E., Okamoto M., Tatematsu K., Yano R., Seo M., Kamiya Y. (2010). Abscisic acid and the control of seed dormancy and germination. Seed Sci. Res..

[29] Bethke P.C., Libourel I.G.L., Jones R.L. (2006). Nitric oxide reduces seed dormancy in *Arabidopsis*. J. Exp. Bot..

[30] Piterková J., Luhová L., Hofman J., Turecková V., Novák O., Petrivalsky M., Fellner M. (2012). Nitric oxide is involved in light-specific responses of tomato during germination under normal and osmotic stress conditions. Ann. Bot..

[31] Lefebvre V., North H., Frey A., Sotta B., Seo M., Okamoto M., Nambara E., Marion-Poll A. (2006). Functional analysis of *Arabidopsis NCED6* and *NCED9* genes indicates that ABA synthesized in the endosperm is involved in the induction of seed dormancy. Plant J..

[32] Hussain A., Mun B.-G., Imran Q.M., Lee S.-U., Adamu T.A., Shahid M., Kim K.-M., Yun B.-W. (2016). Nitric oxide mediated transcriptome profiling reveals activation of multiple regulatory pathways in *Arabidopsis thaliana*. Front. Plant Sci..

[33] Hauser F., Waadt R., Schroeder J.I. (2011). Evolution of abscisic acid synthesis and signaling mechanisms. Curr. Biol..

[34] Szostkiewicz I., Richter K., Kepka M., Demmel S., Ma Y., Korte A., Assaad F.F., Christmann A., Grill E. (2010). Closely related receptor complexes differ in their ABA selectivity and sensitivity. Plant J..

[35] Freschi L. (2013). Nitric oxide and phytohormone interactions: Current status and perspectives. Front. Plant Sci..

[36] Jain P., Bhatla S.C. (2018). Molecular mechanisms accompanying nitric oxide signalling through tyrosine nitration and *S*-nitrosylation of proteins in plants. Funct. Plant Biol..

[37] Castillo M.C., Lozano-Juste J., González-Guzmán M., Rodriguez L., Rodriguez P.L., León J. (2015). Inactivation of PYR/PYL/RCAR ABA receptors by tyrosine nitration may enable rapid inhibition of ABA signaling by nitric oxide in plants. Sci. Signal..

[38] Brock A.K., Willmann R., Kolb D., Grefen L., Lajunen H.M., Bethke G., Lee J., Nürnberger T., Gust A.A. (2010). The *Arabidopsis* mitogen-activated protein kinase phosphatase PP2C5 affects seed germination, stomatal aperture, and abscisic acid-inducible gene expression. Plant Physiol..

[39] Wang P., Zhu J.-K., Lang Z. (2015). Nitric oxide suppresses the inhibitory effect of abscisic acid on seed germination by *S*-nitrosylation of SnRK2 proteins. Plant Signal. Behav..

[40] Oracz K., Karpiński S. (2016). Phytohormones signaling pathways and ROS involvement in seed germination. Front. Plant Sci..

[41] Lozano-Juste J., Leon J. (2010). Enhanced abscisic acid-mediated responses in *nia1nia2noa1-2* triple mutant impaired in NIA/NR- and AtNOA1-dependent Nitric Oxide biosynthesis in *Arabidopsis*. Plant Physiol..

[42] Albertos P., Romero-Puertas M.C., Tatematsu K., Mateos I., Sánchez-Vicente I., Nambara E., Lorenzo O. (2015). *S*-nitrosylation triggers ABI5 degradation to promote seed germination and seedling growth. Nat. Commun..

[43] Johnson R.R., Wagner R.L., Verhey S.D., Walker-Simmons M.K. (2002). The abscisic acid-responsive kinase PKABA1 interacts with a seed-specific abscisic acid response element-binding factor, TaABF, and phosphorylates TaABF peptide sequences. Plant Physiol..

[44] Huang H., Liu B., Liu L., Song S. (2017). Jasmonate action in plant growth and development. J. Exp. Bot..

[45] Yildiz K., Muradoglu F., Yilmaz H. (2008). The effect of jasmonic acid on germination of dormant and nondormant pear (*Pyrus communis* L.) seeds. Seed Sci. Technol..

[46] Ranjan R., Lewak S. (1992). Jasmonic acid promotes germination and lipase activity in non-stratified apple embryos. Physiol. Plant..

[47] Xu Q., Truong T.T., Barrero J.M., Jacobsen J.V., Hocart C.H., Gubler F. (2016). A role for jasmonates in the release of dormancy by cold stratification in wheat. J. Exp. Bot..

[48] Zimmermann G. (2006). The multigene family encoding Germin-Like proteins of barley. Regulation and function in basal host resistance. Plant Physiol..

[49] Han G.-Z. (2016). Evolution of jasmonate biosynthesis and signaling mechanisms. J. Exp. Bot..

[50] Linkies A., Leubner-Metzger G. (2012). Beyond gibberellins and abscisic acid: How ethylene and jasmonates control seed germination. Plant Cell Rep..

[51] Chaki M., Valderrama R., Fernández-Ocaña A.M., Carreras A., López-Jaramillo J., Luque F., Palma J.M., Pedrajas J.R., Begara-Morales J.C., Sánchez-Calvo B. (2009). Protein targets of tyrosine nitration in sunflower (*Helianthus annuus* L.) hypocotyls. J. Exp. Bot..

[52] Fernández-Arbaizar A., Regalado J.J., Lorenzo O. (2012). Isolation and characterization of novel mutant loci suppressing the ABA hypersensitivity of the *Arabidopsis* coronatine insensitive 1-16 (*coi1-16*) mutant during germination and seedling growth. Plant Cell Physiol..

[53] Galland M., Rajjou L. (2015). Regulation of mRNA translation controls seed germination and is critical for seedling vigor. Front. Plant Sci..

[54] Bazin J., Langlade N., Vincourt P., Arribat S., Balzergue S., El-Maarouf-Bouteau H., Bailly C. (2011). Targeted mRNA oxidation regulates sunflower seed dormancy alleviation during dry after-ripening. Plant Cell.

[55] Chomczynski P., Sacchi N. (1987). Single-step method of RNA isolation by acid guanidinium thiocyanate-phenol-chloroform extraction. Anal. Biochem..

